# Endoscopic injection sclerotherapy improves liver function compared with endoscopic variceal ligation

**DOI:** 10.1038/s41598-021-99855-z

**Published:** 2021-10-14

**Authors:** Tsuguru Hayashi, Tatsuyuki Watanabe, Michihiko Shibata, Shinsuke Kumei, Shinji Oe, Koichiro Miyagawa, Yuichi Honma, Masaru Harada

**Affiliations:** Third Department of Internal Medicine, School of Medicine, University of Occupational and Environmental Health, 1-1 Iseigaoka, Yahatanishi-ku, Kitakyushu, 807-8555 Japan

**Keywords:** Gastrointestinal diseases, Gastroenterology, Hepatology

## Abstract

Liver function is a most important prognostic factor in patients with liver cirrhosis. Also, portal hypertension is a fatal complication of liver cirrhosis and variceal treatment is indispensable. However, changes of liver functions after endoscopic variceal treatments are unknown. The aim of this study was to evaluate prognosis and liver functions after endoscopic injection sclerotherapy (EIS) and endoscopic variceal ligation (EVL). A total of liver cirrhotic 103 patients who underwent prophylactic EIS and EVL were enrolled. Overall survival rate was higher in EIS group than EVL group (p = 0.03). Multivariate analysis showed that EIS was a negative factor for death (HR: 0.46, 95% confidence interval: 0.24–0.88, p = 0.02). Liver functions were assessed by blood test taken at before and 3 months after treatment. In EIS group, albumin and prothrombin time improved (p < 0.01), leading to improvement of Child–Pugh score, ALBI score and MELD score (p < 0.05). However, these did not improve in EVL group. EIS was a significant factor related to the elevated value of albumin after treatment in linear regression analysis (estimated regression coefficient: 0.17, 95% confidence interval: 0.05–0.29, p = 0.005). These results revealed that EIS could improve liver functions and prognosis.

## Introduction

Hemorrhage from gastroesophageal varices is one of the most common and serious complications in patients with liver cirrhosis^1^. Although the mortality from variceal hemorrhage has markedly decreased in the last two decades, its overall in hospital mortality is still as high as 16.8%^2^. Therefore, regular endoscopic observation is desirable^3,4^. If the gastroesophageal varices become large, prophylactic endoscopic therapies such as endoscopic injection sclerotherapy (EIS) or endoscopic variceal ligation (EVL) are recommended before the varices rupture^5^.

Several studies have compared EIS with EVL in terms of recurrence, rebleeding and prognosis^6–10^. EIS is superior to EVL in variceal recurrence rate^7,8^. However, EVL is easier to perform and its rate of complication is less than that of EIS^9^. Both endoscopic methods have different strong points. Thus, it is difficult to determine which prophylactic treatment should be selected.

Liver functions is an important factor that influences prognosis in patients with advanced chronic liver disease^11^. In patients with hepatocellular carcinoma (HCC), treatment options are limited depending on the liver functions^12–14^. However, no study have compared EIS with EVL from the viewpoint of liver functions. Clarification of liver functions after EIS and EVL is therefore an issue of major importance.

We researched prognosis and long-term changes of liver functions after prophylactic endoscopic therapies for gastroesophageal varices. The aim of this study was to compare EIS with EVL in regard to prognosis and changes of liver functions after treatments.

## Results

### Patient characteristics

A total of 127 patients underwent prophylactic EIS and/or EVL. Among them, patients with no follow up (n = 17) and incomplete data (n = 7) were excluded from the study. Therefore, a total of 103 patients were enrolled in this study. The number of patients in the EIS and EVL groups was 64 and 39, respectively. The variceal forms were F2 or F3, and red wale sign was positive in all patients. Their baseline characteristics were shown in Table1. No factors differed between the two groups. All patients were performed as primary prophylaxis for variceal bleeding. No patients started nucleotide analogs for hepatitis B virus (HBV) and direct-acting antiviral agents for hepatitis C virus (HCV) within 3 months after endoscopic treatments.

**Table 1:** Baseline characteristics in EIS and EVL group.

	EIS (n = 64)	EVL (n = 39)	p value
Age, years	66 (28 to 85)	67 (43 to 87)	0.68
Male (%)	68.8	64.1	0.67
BMI, kg/m^2^	23.2 (18.1 to 38.9)	23.1 (16.7 to 37.9)	0.77
Etiology (HBV/HCV/NBNC)	12/15/37	2/12/25	0.14
Albumin, g/dL	3.5 (2.5 to 4.2)	3.5 (2.3 to 4.2)	0.32
Bilirubin, mg/dL	1.1 (0.3 to 2.9)	1.2 (0.3 to 3.1)	0.052
AST, U/L	39 (17 to 90)	42 (13 to 91)	0.80
ALT, U/L	28 (10 to 86)	28 (6 to 86)	0.89
Cre, mg/dL	0.76 (0.43 to 1.42)	0.80 (0.41 to 4.26)	0.55
eGFR, mL/min/1.73 m^2^	70.7 (31.1 to 161)	69.0 (13.0 to 119)	0.52
PT, %	69.9 (38.7 to 97.9)	68.6 (47.5 to 100.0)	0.97
WBC, /μL	3900 (1200 to 8100)	3700 (1100 to 6600)	0.08
HGB, g/dL	11.8 (7.0 to 13.0)	11.3 (7.1 to 14.3)	0.19
PLT, × 10^4^/μL	8.9 (2.6 to 20.3)	8.4 (2.5 to 26.1)	0.41
Child–Pugh score	7 (5 to 9)	7 (5 to 9)	0.36
Fib-4 Index	5.5 (2.0 to 21.8)	6.4 (1.5 to 19.2)	0.27
ALBI score	− 2.18 (− 2.82 to − 1.28)	− 2.09 (− 2.79 to − 0.89)	0.13
MELD score	6 (− 1 to 13)	6 (2 to 21)	0.22
Past history of HCC (%)	31.2	41.0	0.40
Use of non-selective beta blockade (%)	7.8	5.1	0.71

p values are results that compared EIS group with EVL group. Categorical variables were analyzed using χ^2^-test or Fisher’s exact test, and continuous variables were compared using Mann–Whitney’s *U* test. alfa fetoprotein, *ALBI* albumin–bilirubin, *AST* aspartate transaminase, *ALT* alanine aminotransferase, *BMI* body mass index, *Cre* creatinine, *eGFR* estimated glomerular filtration rate, *EIS* endoscopic injection sclerotherapy, *EVL* endoscopic variceal ligation, *HBV* hepatitis B virus, *HCC* hepatocellular carcinoma, *HCV* hepatitis C virus, *HGB* hemoglobin, *MELD* the model for end-stage liver disease, *NBNC* non-hepatitis B virus and non-hepatitis C virus, *PLT* platelet, *PT* prothrombin time, *WBC* white blood cell.

### Prognosis after prophylactic variceal treatment

**Figure 1:**
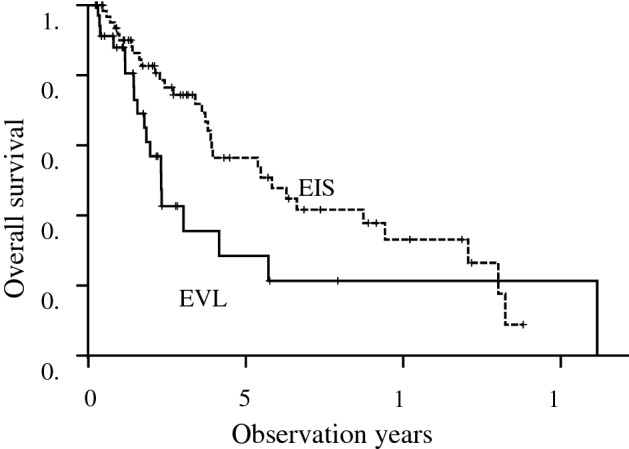
Overall survival in patients in the EIS group (dotted line) and EVL group (solid line). The EIS group showed a better prognosis than the EVL group (5 years survival rate: 56.5% vs 28.4%, p = 0.03).

The median follow-up period of the 103 patients was 2.2 (0.3–16.2) years and 5 years overall survival rate was 47.9%. Stratified to method of treatments, 5 years survival rate of the EIS group was significantly longer than that of the EVL group (56.5% vs 28.4%, p = 0.03) (Fig. 1). In univariate analysis, age, etiology, Child–Pugh grade B, EIS, and past history of HCC were significant influencing factors for death. In multivariate analysis, Child–Pugh grade B (HR: 2.72, 95% confidence interval (CI): 1.39–5.32, p = 0.004), EIS (HR: 0.46, 95% CI: 0.24–0.88, p = 0.02) and past history of HCC (HR: 2.33, 95% CI: 1.20–4.51, p = 0.01) were significant influencing factors for death (Table 2).

**Table 2:** Cox regression analysis about baseline factors associated with all-cause death in all patients.

	Univariate analysis			Multivariate analysis		
	HR	95% CI	p-value	HR	95% CI	p-value
**Age, years**						
≤ 65	1			1		
> 65	1.85	1.04–3.32	0.04	1.75	0.93–3.29	0.08
**Gender**						
Male	1					
Female	1.24	0.67–2.29	0.50			
**BMI**						
≤ 23.8	1					
> 23.8	1.01	0.56–1.79	0.99			
**Etiology**						
HBV	1			1		
HCV	3.08	1.02–9.34	0.047	2.28	0.72–7.19	0.16
NBNC	1.82	0.63–5.22	0.27	1.57	0.49–5.07	0.45
**Child–Pugh grade**						
A	1			1		
B	1.66	1.30–2.13	< 0.001	2.72	1.39–5.32	0.004
**Treatment**						
EVL	1			1		
EIS	0.52	0.29–0.95	0.03	0.46	0.24–0.88	0.02
**Past history of HCC**						
No	1			1		
Yes	2.86	1.59–5.14	< 0.001	2.33	1.20–4.51	0.01

*BMI* body mass index, *CI* confidence interval, *EIS* endoscopic injection sclerotherapy, *HBV* hepatitis B virus, *HCC* hepatocellular carcinoma, *HCV* hepatitis C virus, *NBNC* non-hepatitis B virus and non-hepatitis C virus.

Conversely, 3 years rebleeding or retreatment rate was not different between the two groups (EIS: 32.5% vs EVL: 26.7%, p = 0.18).

### Liver function after prophylactic endoscopic variceal treatment

Albumin and prothrombin time improved significantly after treatment in the EIS group (p < 0.01), but not in the EVL group (Table 3). In the EIS group, Child–Pugh score, ALBI score and MELD score significantly improved after 3 months. In contrast, albumin and prothrombin time did not improve in the EVL group. Other liver functions, renal functions and blood count were not changed significantly in EIS and EVL groups.

**Table 3:** Liver functions at baseline and 3 months after variceal treatments.

	EIS group			EVL group		
	Baseline	After treatment	p value	Baseline	After treatment	p value
Albumin, g/dL	3.5 (2.5 to 4.2)	3.6 (2.7 to 4.5)	0.002	3.5 (2.3 to 4.2)	3.4 (1.9 to 4.2)	0.17
Bilirubin, mg/dL	1.1 (0.3 to 2.9)	1.1 (0.3 to 4.7)	0.87	1.2 (0.3 to 3.1)	1.2 (0.5 to 3.0)	0.82
AST, IU/L	39 (17 to 90)	39 (20 to 104)	0.81	42 (13 to 91)	42 (17 to 93)	0.19
ALT, IU/L	28 (10 to 86)	27 (6 to 92)	0.49	28 (6 to 86)	26 (7 to 132)	0.48
Cre, mg/dL	0.76 (0.43 to 1.42)	0.71 (0.46 to 1.48)	0.12	0.77 (0.41 to 4.26)	0.76 (0.41 to 1.00)	0.24
eGFR, mL/min/1.73 m^2^	70.7 (31.1 to 161)	73.0 (36.7 to 157)	0.09	69.7 (13.0 to 119)	64.7 (11.0 to 116)	0.33
PT, %	69.9 (38.7 to 97.9)	70.5 (12 to 100)	< 0.001	68.6 (47.5 to 100.0)	65.2 (31 to 100)	0.38
Child–Pugh score	7 (5 to 9)	6 (5 to 11)	0.02	7 (5 to 9)	7 (5 to 11)	0.040
ALBI score	− 2.18 (− 2.82 to − 1.28)	− 2.34 (− 2.99 to − 1.04)	0.003	− 2.09 (− 2.79 to − 0.89)	− 1.99 (− 2.73 to − 0.53)	0.069
MELD score	6 (− 1 to 13)	6 (− 1 to 22)	0.04	6 (2 to 21)	6 (2 to 23)	0.43

*ALBI* albumin–bilirubin, *ALT* alanine aminotransferase, *AST* aspartate transaminase, *Cre* creatinine, *eGFR* estimated glomerular filtration rate, *EIS* endoscopic injection sclerotherapy, *EVL* endoscopic variceal ligation, *MELD* the model for end-stage liver disease, *PT* prothrombin time.

**Figure 2:**
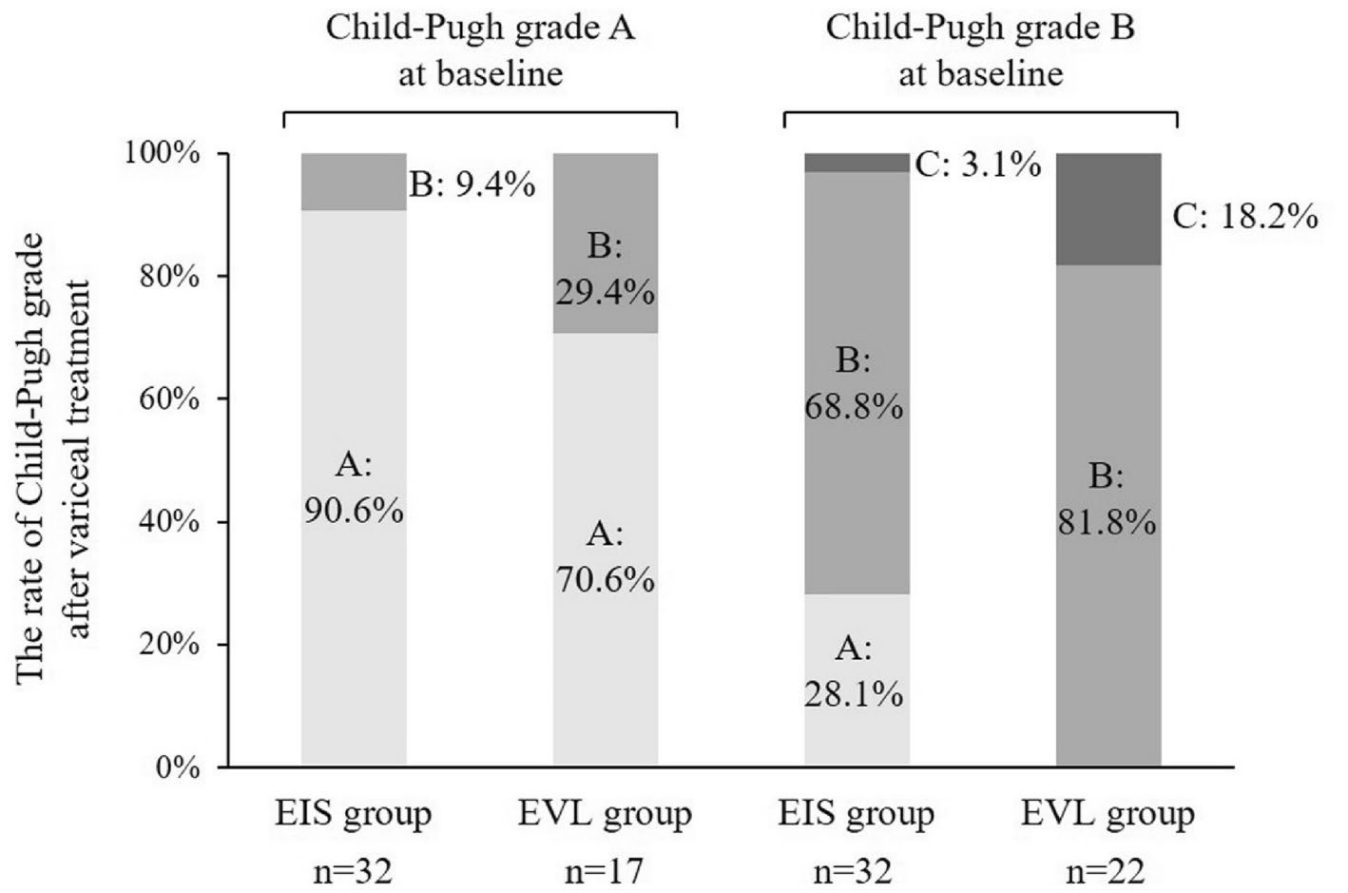
Change of Child–Pugh grade before and after variceal treatments. In patients with Child–Pugh grade A, the rate of maintaining Child–Pugh grade A was higher in the EIS group (90.6 vs 70.6%, p = 0.11). In patients with Child–Pugh grade B, the rate of improving to Child–Pugh grade A was significantly higher in the EIS group (28.1 vs 0.0%, p = 0.015).

The rate of change in Child–Pugh grade after variceal treatment is shown in Fig. 2. Among patients with Child–Pugh grade A at baseline, 90.6% (n = 29) in the EIS group and 70.6% (n = 12) in the EVL group maintained Child–Pugh grade A, respectively. More importantly, 28.1% of patients of Child–Pugh grade B (n = 32) improved to Child–Pugh grade A (n = 9). The rate of Child–Pugh improvement from B to A was significantly higher in the EIS group (p = 0.015).

In linear regression analysis of elevated albumin levels after variceal treatments, EIS was a single independent predictor for improvement of albumin (estimated regression coefficient: 0.17, 95% CI: 0.05–0.29, p = 0.005) (Table 4). Other factors were not significant.

**Table 4:** Linear regression analysis about factors associated with increasing of serum albumin levels after variceal treatment in all patients.

	Estimated regression coefficient	95% CI	p-value
Age, years (> 65)	− 0.02	− 0.14 to 0.10	0.79
Gender (male)	− 0.06	− 0.19 to 0.06	0.33
BMI (> 23.8)	0.02	− 0.10 to 0.15	0.72
Etiology (NBNC)	0.03	− 0.09 to 0.15	0.62
Child–Pugh (B)	0.02	− 0.10 to 0.13	0.80
Treatment (EIS)	0.17	0.05 to 0.29	0.005
Past history of HCC (present)	− 0.08	− 0.21 to 0.04	0.18

*BMI* body mass index, *CI* confidence interval, *EIS* endoscopic injection sclerotherapy, *EVL* endoscopic variceal ligation, *HBV* hepatitis B virus, *HCC* hepatocellular carcinoma, *HCV* hepatitis C virus, *NBNC* non-hepatitis B virus and non-hepatitis C virus.

## Discussion

We first showed that the prognosis of EIS group was significantly better than EVL group. Second, we demonstrated that prophylactic EIS for gastroesophageal varices could significantly improve liver functions, whereas EVL did not improve liver functions. In particular, the rate of patients whose liver function increased from Child–Pugh grade B to A was higher in EIS group. In addition, linear regression analysis revealed that elevated albumin level after variceal treatment was associated with EIS.

A large number of studies have compared EIS with EVL^6–10^. EIS occludes the blood flow that supplies esophageal varices with sclerosing agent. This is the reason why EIS shows a lower recurrence rate of gastroesophageal varices than EVL^7,9^. Therefore, EIS is selected as a first choice of prophylactic variceal treatment in Japan. However, prognosis after variceal treatments and factors associated with prognosis are still unknown. Therefore, we studied the prognosis and liver functions after variceal treatments. This is the first study that compared EIS with EVL from the viewpoint of liver functions. Our study showed that the course of liver functions differed after these two prophylactic variceal treatments. This could lead to significant better prognosis after EIS than EVL.

Hepatic blood flow gradually decreases with progression of chronic liver disease, leading to decline in hepatic functions^15–17^. In particular, portal blood flow gradually decreases in inverse proportion to Child–Pugh class and indocyanine green test^18,19^. That results in decreasing transport of glucose, amino acid and fatty acid to hepatocytes and an increase in oxidative stress and liver inflammation. In response to decreased portal flow, hepatic arterial flow increases complementarily^20,21^. However, in hyperdynamic state, superior mesenteric artery and splenic arterial flow are increased and result in an increase of blood flow in the portal system. These hemodynamics causes development of collateral vessels, especially emergence of gastroesophageal varices. However, EIS changes the hepatic hemodynamics by occluding collateral blood flow. Takahashi et al. reported that portal venous flow increased after EIS^22^. The increase of blood flow in the liver sinusoid increases shear stress, which causes the release of a variety of cytokines such as interleukin (IL)-6, hepatocyte growth factor and nitric oxide from the sinusoidal endothelium^23–25^. These could induce hepatocyte proliferation. In fact, after portal vein embolization (PVE), which is a preoperative preparation for extensive liver resection, the volume of the non-embolized lobe was increased and the Ki-67 labelling index was higher in the non-embolized lobe^26^. These evidences suggest that the number of hepatocytes increased and liver regeneration occurred by repairing portal hemodynamics. This may lead improvement of liver functions.

The same mechanism is shown in balloon occluded retrograde transvenous obliteration (BRTO) and percutaneous transhepatic obliteration (PTO). Several previous studies reported that BRTO could increase portal venous flow and improve liver functions by obstruction of the portosystemic shunt^27,28^. However, not all patients who underwent BRTO showed improved liver functions^29^. Also in our study, some patients did not experience improved liver functions. The reason for poor response to increased portal flow is uncertain. We have to clarify this problems and other predictive factors for improvement of liver functions.

We demonstrated that EIS improved liver functions. However, we have to mention that patients with Child–Pugh grade C were not enrolled in this study. Patients with uncontrollable ascites or hyperbilirubinemia were not indicated for prophylactic EIS. When liver damage is advanced, EIS could cause liver failure and death in several days^30^. Therefore, it is important to consider appropriate indications for EIS.

This study has several limitations. First, we carried out this study with a small sample size. This could have an impact on statistics. Second, the present study was analyzed retrospectively. Third, selection of treatment method was not randomized. EVL may be performed in patients with poor general conditions. However, there was no difference between EIS and EVL groups at baseline in this study. Therefore, a prospective study with large number of patients should be performed to analyze prophylactic variceal treatments.

In conclusion, we demonstrated that EIS has the potential to improve liver functions, which could lead to a better prognosis than with EVL.

## Methods

### Patients

We retrospectively analyzed the liver cirrhotic patients who underwent prophylactic EIS and/or EVL from April 2002 to July 2020 in our hospital. The diagnosis of gastroesophageal varices was based on endoscopic findings at 1 month before variceal treatment. Endoscopic findings of esophageal varices were evaluated according to the grading system defined by the Japanese Research Committee on Portal Hypertension and outlined in the general rules for recording endoscopic findings of esophageal varices^31^. Varices were classified as F1: small and straight, F2: enlarged and tortuous, or F3: large and coil-sharped. EIS was performed on red wale sign positive or F2/F3 variceal patients. Patients with the following conditions were excluded from the study; (1) lost to follow up and (2) incomplete data. Patients with Child–Pugh grade C or major portal vein tumor thrombus were not included in this study, because they were not recommended for prophylactic variceal treatment. Regarding the selection of variceal treatments, EIS was our first choice and we changed perform EVL if it was difficult to do intravariceal injection. This study was conducted in accordance with the 1975 Declaration of Helsinki. This study was approved by the institutional review board of our hospital (the ethics committee name is ethics committee of university of occupational and environmental health, Japan and the code number is H29-079). This study is a retrospective observational study and gives no disadvantage to patients. Therefore, the ethics committee decided that informed consent is not required and waived. However, we announced publicly that patients could refuse to participate in this study if they desire.

## Endoscopic treatment

EIS was performed using a flexible gastrointestinal endoscope (GIF Q260J: Olympus Optical, Tokyo, Japan) under fluoroscopy and a combination of intermittent intravariceal injection of 5% ethanolamineoleate with iopamidol (5% EOI). Oral side of the injection point was occluded by balloon, and we injected EOI retrogradely to supplying vessels. EIS was repeated weekly until disappearance of variceal form and red wale sign. When it was difficult to perform intravariceal injection, EVL was added.

EVL was performed using pneumo-activate EVL device (Sumius, Tokyo, Japan) and cylinders. Ligation bands were applied to varices in a step ladder pattern. This procedure was also repeated until disappearance of variceal form and red wale sign.

Argon plasma coagulation (APC) was added in both groups after EIS or EVL according to the judgement of endoscopic specialists.

### Prognosis and liver function assessment

Clinical and laboratory information of patients was obtained from electronic medical records. Medical historical variables consisted of age, sex, body mass index (BMI), etiology of liver cirrhosis, Child–Pugh score and grade^32^, Fib-4 index^33^, ALBI score^34^, MELD score^35^, history of HCC and use of non-selective beta blockade. Overall survival (OS) and time to rebleeding or retreatment were compared between EIS and EVL groups. OS was duration time from the variceal treatment to death from any cause or last follow-up. Blood variables included liver functions test (serum total bilirubin, albumin, aspartate aminotransferase, alanine aminotransferase, gamma-glutamyl transpeptidase, prothrombin time), renal functions (serum creatinine, estimated glomerular filtration rate) and complete blood count at baseline and 3 months after endoscopic treatment.

### Statistical analysis

Baseline parameters were compared between the EIS and EVL groups using Mann–Whitney *U* test and χ^2^ test. Baseline data comparing the EIS group and EVL group were shown as median value (minimum to maximum values). The Kaplan–Meier method with log rank test was used to analyze the prognosis and the Cox proportional hazard model was used for univariate and multivariate analysis of factors for prognosis. p values were calculated for all tests, with a value of p < 0.05 considered to be statically significant. Changes of liver functions after EIS or EVL were compared using the Wilcoxon single-rank sum test and Fisher’s exact test. Linear regression analyses were performed to identify the factors associated with elevated serum albumin levels. We defined change of serum albumin levels as differences from baseline to 3 months after treatment. All statistical analyses were performed using the Statistical Package for the Social Science (SPSS) version 25 (SPSS Inc., Chicago, IL, USA) and Easy R (EZR) version 1.29 (Saitama Medical center, Jichi Medical University, Saitama, Japan), and graphical use interface for R (The R Foundation for Statistical Computing, Vienna, Austria)^36^.

## Data Availability

All relevant data are within the manuscript.
